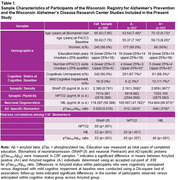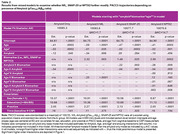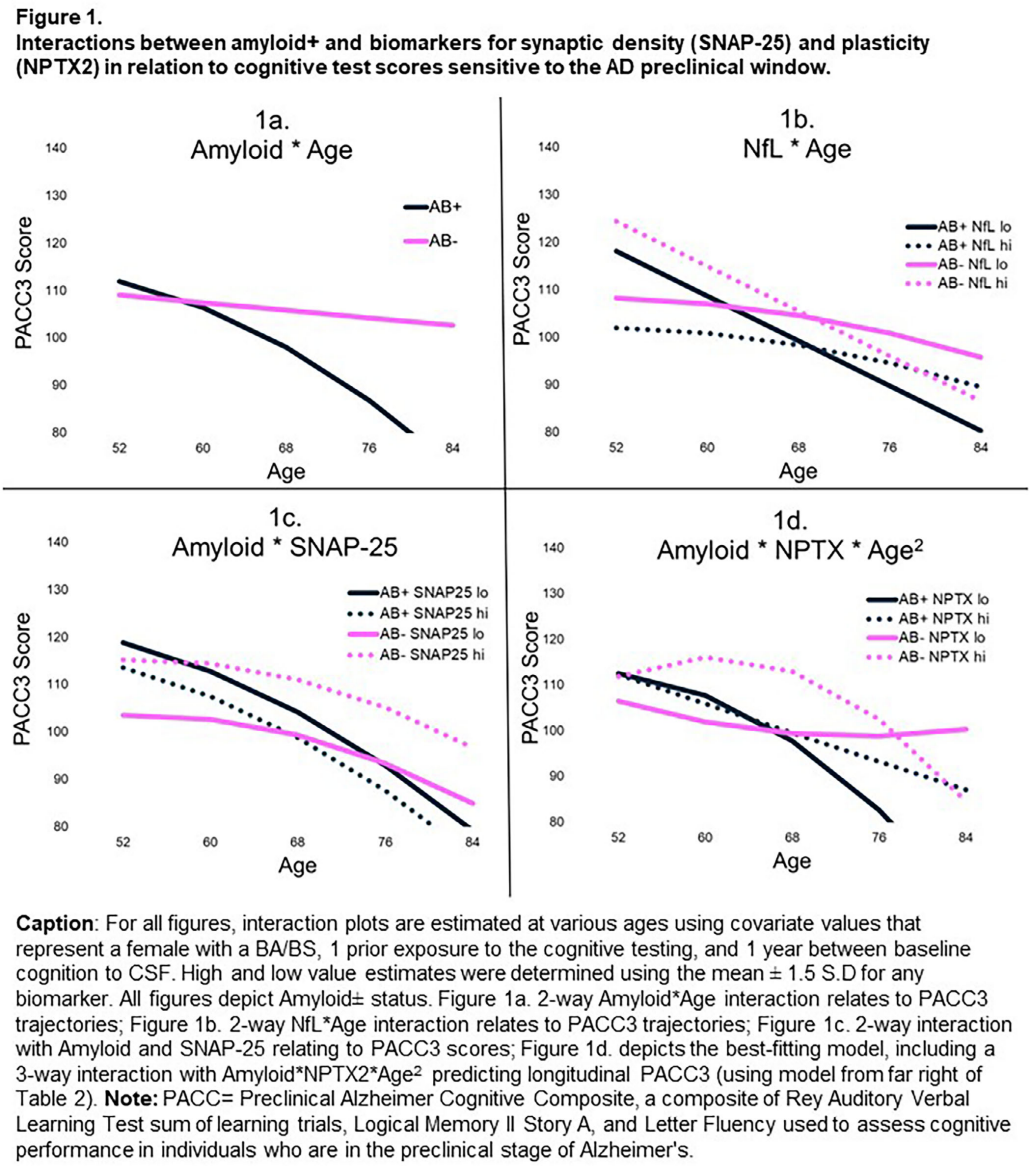# CSF SNAP‐25 and Neuronal pentraxin 2 associations with cognitive decline in the presence of CSF ptau181/Aβ42 in an at‐risk cohort

**DOI:** 10.1002/alz.091494

**Published:** 2025-01-09

**Authors:** Beth M. Planalp, Rebecca E. Langhough, De'Karlos Valentino, Denise Ealy, Clara Quijano‐Rubio, Gwendlyn Kollmorgen, Kaj Blennow, Henrik Zetterberg, Erin M. Jonaitis, Sterling C. Johnson

**Affiliations:** ^1^ University of Wisconsin‐Madison, Madison, WI USA; ^2^ Roche Diagnostics International Ltd., Rotkreuz Switzerland; ^3^ Roche Diagnostics GmbH, Penzberg Germany; ^4^ Clinical Neurochemistry Laboratory, Sahlgrenska University Hospital, Mölndal Sweden; ^5^ Department of Psychiatry and Neurochemistry, Institute of Neuroscience and Physiology, the Sahlgrenska Academy at the University of Gothenburg, Mölndal Sweden; ^6^ Department of Neurodegenerative Disease, UCL Queen Square Institute of Neurology, University College London, London, ‐ UK; ^7^ UK Dementia Research Institute, University College London, London UK

## Abstract

**Background:**

Alzheimer's disease (AD) is identified by the accumulation of amyloid β (Aβ) and tau proteins in the brain. The NeuroToolKit offers automated cerebrospinal fluid (CSF) immunoassays of core AD biomarkers and biomarkers of neurodegeneration and synaptic function, including neurofilament light (NfL), SNAP‐25, and neuronal pentraxin 2 (NPTX2). This work explores whether these three markers predict pre‐dementia cognitive decline synergistically with or after accounting for CSF ptau_181_/Aβ_42_.

**Method:**

Participants included N=360 baseline dementia‐free adults (45—85 years) with complete CSF biomarker and cognitive data from the Wisconsin Registry for Alzheimer’s Prevention and the Wisconsin Alzheimer’s Disease Research Center (on‐going, longitudinal preclinical AD studies). We assessed CSF biomarkers using the NeuroToolKit (NTK), a panel of robust prototype biomarker assays (Roche Diagnostics International, Switzerland). Mixed models examined separately whether NfL, SNAP‐25, or NPTX2 biomarkers further modified Preclinical Alzheimer’s Cognitive Composite (PACC3) trajectories beyond Aβ+ effects. Biomarker*ptau_181_/Aβ_42_*age (linear and quadratic) interactions were examined, and non‐significant higher order interactions removed sequentially before interpreting NfL, SNAP‐25, and NPTX2 effects.

**Result:**

Sample characteristics are shown in Table 1, and show significant correlations among all biomarker pair except NPTX2 and ptau_181_/Aβ_42_. Table 2 and Figure 1 depict PACC3 model outputs and biomarker associations; Aβ+ performed worse or declined faster than Aβ‐ across all models. Compared to a base “amyloid‐only” model, NfL, SNAP‐25, and NPTX2 “biomarker” models showed an improved model fit (Table 2). The best‐fitting model included the NPTX2* ptau_181_/Aβ_42_*age^2^ interaction (Table 2) which showed greatest average PACC3 decline in those with higher amyloid and lower NPTX2 (Figure 1d). SNAP‐25 also related to cognition such that individuals with lower amyloid and higher SNAP‐25 had the highest PACC3 average (interactions with age were non‐significant), and individuals with higher NfL showed steeper PACC3 decline than those with lower NfL.

**Conclusion:**

In addition to known effects of NfL, recently developed NTK immunoassays for SNAP‐25 and NPTX2 accounted for additional variability on AD‐related pre‐dementia PACC3 performance. Future work may identify clinically useful cutpoints for SNAP‐25 and NPTX2 and explore relations with other biomarkers common to neurodegeneration and AD. We will also examine associations with other outcomes including clinical progression of AD.